# Insights into the Effect of Chitosan and β-Cyclodextrin Hybridization of Zeolite-A on Its Physicochemical and Cytotoxic Properties as a Bio-Carrier for 5-Fluorouracil: Equilibrium and Release Kinetics Studies

**DOI:** 10.3390/molecules28145427

**Published:** 2023-07-15

**Authors:** Mashael D. Alqahtani, May N. Bin Jumah, Saleha A. AlZahrani, Ahmed A. Allam, Mostafa R. Abukhadra, Stefano Bellucci

**Affiliations:** 1 Department of Biology, College of Science, Princess Nourah bint Abdulrahman University, P.O. Box 84428, Riyadh 11671, Saudi Arabia; mdalqahtani@pnu.edu.sa (M.D.A.);; 2 Zoology Department, Faculty of Science, Beni-Suef University, Beni-Suef 65211, Egypt; 3 Geology Department, Faculty of Science, Beni-Suef University, Beni-Suef 65211, Egypt; 4 Materials Technologies and Their Applications Lab, Geology Department, Faculty of Science, Beni-Suef University, Beni-Suef 65211, Egypt; 5 INFN-Laboratori Nazionali di Frascati, Via E. Fermi 54, 00044 Frascati, Italy

**Keywords:** zeolite-A, biopolymers, composites, 5-fluorouracil, loading, cytotoxicity

## Abstract

Synthetic zeolite-A (ZA) was hybridized with two different biopolymers (chitosan and β-cyclodextrin) producing biocompatible chitosan/zeolite-A (CS/ZA) and β-cyclodextrin/zeolite-A (CD/ZA) biocomposites. The synthetic composites were assessed as bio-carriers of the 5-fluorouracil drug (5-Fu) with enhanced properties, highlighting the impact of the polymer type. The hybridization by the two biopolymers resulted in notable increases in the 5-Fu loading capacities, to 218.2 mg/g (CS/ZA) and 291.3 mg/g (CD/ZA), as compared to ZA (134.2 mg/g). The loading behaviors using ZA as well as CS/ZA and CD/ZA were illustrated based on the classic kinetics properties of pseudo-first-order kinetics (R^2^ > 0.95) and the traditional Langmuir isotherm (R^2^ = 0.99). CD/ZA shows a significantly higher active site density (102.7 mg/g) in comparison to CS/ZA (64 mg/g) and ZA (35.8 mg/g). The number of loaded 5-Fu per site of ZA, CS/ZA, and CD/ZA (>1) validates the vertical ordering of the loaded drug ions by multi-molecular processes. These processes are mainly physical mechanisms based on the determined Gaussian energy (<8 kJ/mol) and loading energy (<40 kJ/mol). Both the CS/ZA and CD/ZA 5-Fu release activities display continuous and controlled profiles up to 80 h, with CD/ZA exhibiting much faster release. According to the release kinetics studies, the release processes contain non-Fickian transport release properties, suggesting cooperative diffusion and erosion release mechanisms. The cytotoxicity of 5-Fu is also significantly enhanced by these carriers: 5-Fu/ZA (11.72% cell viability), 5-Fu/CS/ZA (5.43% cell viability), and 5-Fu/CD/ZA (1.83% cell viability).

## 1. Introduction

Around 72% of all documented fatalities globally are caused by non-contagious illnesses, mainly cancer, and that percentage is predicted to increase to 75% over the next several years [1,2]. Colorectal cancer is a prevalent malignant kind of cancer that affects around 13% of cancer patients globally and is one of the two main causes of mortality, raising the global mortality rate [3,4]. Colorectal cancer starts as a polyp inside the mucosal layers and expands to the present submucosa and neighboring tissues. The generated neoplastic cells migrate into the surrounding organs and lymphatic nodes in the latter phases of colorectal cancer [5]. Therefore, the development of efficient and safe therapies that are capable of inhibiting tumor cells without discernible severe negative side effects represents an urgent challenge and an active area of inquiry in the medical and scientific communities [6,7]. 

Chemotherapies of many types are frequently employed to combat cancer cells’ growth and proliferation [8,9]. The frequently used chemotherapies produce a considerable amount of oxidative stress and successfully inhibit DNA replication, which destroys the cancerous cells [6,10]. Unfortunately, the majority of currently used chemotherapies have toxic impacts on normal cells and have numerous significant side effects, particularly at high dosages, including nausea, kidney failure, and bone marrow suppression. Consequently, a number of studies have been developed for enhancing the safety, biocompatibility, curative properties, and specificity of the known types of conventional chemotherapies [11]. This enhancement has been proposed to be accomplished by either producing novel forms of anticancer medicines or by increasing the effectiveness and biosafety of commercially available conventional forms [3].

5-Fluorouracil (5-Fu) is one of the most commonly used drugs in chemotherapy during the treatment of different types of tumor cells, such as those in the rectum, breast, colorectal, and stomach cancers [1,12]. Unfortunately, like most chemotherapies, the application of 5-FU is associated with several drawbacks that are related to its limited solubility, low selectivity, and high diffusion rate, in addition to the reported significant toxic properties of too high a dosage [13,14]. 5-Fu exhibits strong toxic effects on the neural, gastrointestinal, cardiac, hematological, and dermatological systems [12,15]. As a result, several advanced delivery systems have been studied as effective methods to improve the therapeutic activity, curative value, solubility, release rate, and selectivity of 5-Fu [1,11,16]. The effective encapsulation of the drug molecules into advanced biocompatible carriers was strongly recommended to regulate the delivered dosages at certain intervals and at controlled rates to avoid the commonly reported health drawbacks and expand the interaction duration [17,18,19]. Moreover, this can significantly enhance patient compliance and curative profiles in addition to its reduction effect on the degradation rate of the drug, which preserves its concentration at the recommended level [18,19].

In this regard, zeolite, mesoporous silica, polymers, CaO, layered double hydroxide (LDH), and montmorillonite, in addition to different types of organic and inorganic materials, have been assessed as potential carriers of conventional chemotherapies [4,6,11,20,21]. The earlier materials showed a considerable positive influence on the drug’s permeability and retention characteristics [4,5,6,9,17]. Zeolite, as well as silicate materials and their related structures, particularly the synthetic phases, have distinctive characteristics that enable them to be blended into composites with various materials and used as drug carriers for numerous species [4,11,22]. Its structure has a high degree of biocompatibility, a large surface area, is non-toxic, and has a high adsorption capacity, chemical reactivity, ion exchange characteristics, and structural flexibility [23]. Zeolite materials, like other aluminosilicate materials, have strong hydrophilic characteristics that diminish their affinity for the organic ions of the drugs and, as a result, their loading capacities [24]. It has been observed that polymeric modification of zeolite materials improves their organophilicity as well as their pore size distribution, thereby promoting their loading and release characteristics [18,25].

Chitosan is a widely recognized biopolymer that plays a crucial role in various pharmaceutical, environmental, and medical applications, particularly as a drug carrier [26,27,28]. Chitosan is a polyaminosaccharide polymer that has significant technical advantages and may be easily manufactured from the chitin component in various biogenic sources [26]. Technically, chitosan chains have significant safety, hemostatic ability, bioactivity, antimicrobial capacity, biocompatibility, and biodegradability, in addition to high mechanical and adsorption properties [28]. Furthermore, β-cyclodextrin (β-CD) polymer is a widely used and significant biopolymer that has been extensively investigated as an essential component in several composites with a variety of inorganic materials for different medicinal and environmental applications [1,29]. This was primarily attributed to its high accessibility, excellent biocompatibility, marked chemical stability, non-toxicity, and significant adsorption characteristics [30,31]. Chemically, β-CD has a cyclic glucopyranose structure consisting of between six and seven units of glucose joined or linked to each other via different kinds of (1→4) glycosidic bonds [25,29]. While the exterior surface of the structural units of β-CD show substantial polarity, the interior structure possesses hydrophobic characteristics [28]. This greatly encourages its incorporation into composites that consist of inorganic materials and improves the synthetic carriers’ potential to load drugs depending on their structural characteristics [32,33]. As a component in a delivery system, the β-CD considerably increases the drug’s physicochemical characteristics, including its physical stability, solubility in water, therapeutic effectiveness, and chemical stability [34,35].

Therefore, this study aims to follow the impact of the surface modifications of synthetic zeolite-A with chitosan (CS/ZA) and β-cyclodextrin (CD/ZA) biopolymers on the properties of the obtained biocomposites as delivery systems for the 5-fluorouracil drug. The potential of the composites as delivery systems was assessed based on a detailed investigation of the loading properties and the controlling mechanisms. Furthermore, the release properties and the release kinetics were presented and discussed in the study, in addition to the cytotoxicity properties of the studied materials as anticancer agents against colorectal cancer cells (HCT-116). 

## 2. Results and Discussion 

### 2.1. Characterization of the Carrier

#### 2.1.1. XRD Analysis

**Figure 1:**
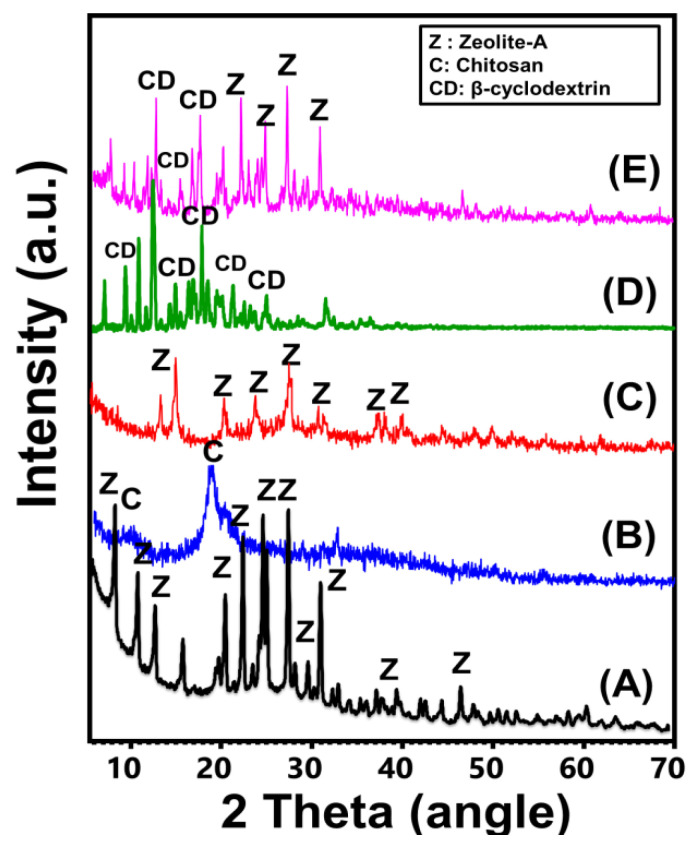
XRD patterns of zeolite-A (**A**), chitosan (**B**), CS/ZA composite (**C**), β-CD polymer (**D**), and synthetic CD/ZA composite (**E**).

The synthetic zeolite incorporated during the production of the two composites exhibits the characteristic XRD pattern of synthetic zeolite-A (Figure 1). The identification peaks were observed clearly at 7.2°, 10.32°, 12.6°, 16.2°, 21.83°, 24°, 26.2°, 27.2°, 30.1°, 30.9°, 31.1°, 32.60°, 33.39°, and 34.3° [36] (Figure 1A). Regarding the observed pattern of integrated chitosan, it reflects the semi-crystalline properties of commercial chitosan, with two broad peaks around 9.91° and 20.22° [26] (Figure 1B). The recognized pattern of the synthetic CS/ZA reflects significant interaction between the two components of the composite. Several identifiable peaks of zeolite were reduced strongly in addition to deviations in their positions (15.48°, 21.65°, 25.52°, 27.11°, and 30.6° (Figure 1C)). For the β-CD polymer, the observed pattern demonstrates the characteristic peaks of commercially used crystalline β-CD polymer (6.8°, 9.21°, 10.83°, 12.57°, 12.64°, 12.8°, 13.0°, 15.55°, 18.9°, 19.8°, 21.90°, 23°, 25.80°, 27.34°, 30.30°, and 34.90°) (Figure 1D). Regarding the hybridized zeolite-A with β-CD, the observed patterns validate complex peaks related to both of the components but with noticeable shifting in their positions (Figure 1E). The residual peaks of zeolite-A were detected at 7.25°, 10.26°, 12.50°, 16.16°, 21.75°, 24.0°, 26.70°, 27.2°, 30.0°, 30.96°, and 32.66° while the identified residual peaks were 8.94°, 10.26°, 13.07°, 18.83°, 20.9°, and 26.19° (Figure 1E). 

#### 2.1.2. SEM and HRTEM Analyses

**Figure 2:**
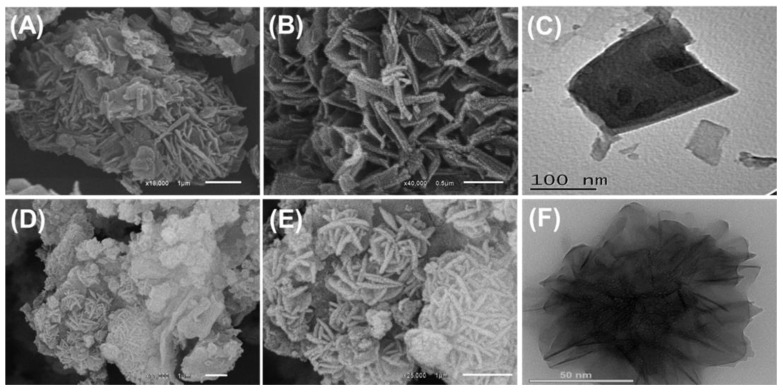
SEM images of CS/ZA composite (**A**,**B**), HRTEM images of CS/ZA composite (**C**), SEM images of synthetic CD/ZA composite (**D**,**E**), and HRTEM images of synthetic CD/ZA composite (**F**).

The incorporated synthetic zeolite exhibits the characteristic cubic morphology of synthetic zeolite-A in SEM images (Figure 1). Regarding the integrated CS/ZA, the obtained SEM images show remarkable changes in the surficial morphology (Figure 2A,B). The ZA grains were highly coated with the incorporated chitosan polymer, and the high-magnification images demonstrate the existence of the polymeric matrix as intersected rod-like or fiber-like particles, including a secondary nanoporous matrix (Figure 2A,B). The HRTEM images of CS/ZA are in agreement with the marked morphologies in the SEM images (Figure 2C). The cubic grains of ZA are observed as inclusions within the matrix of the chitosan polymer (Figure 2C). Moreover, the intersection between the chitosan rod-like particles is clearly detected, as well as the formation of a porous matrix.

Such morphological changes are also observed for CD/ZA; the formed particles display noticeable agglomeration properties (Figure 2D,E). The zeolite grains in the agglomerated CD/ZA particles exhibit significant random re-orientation, which results in remarkable rugged surficial features (Figure 2D). These grains, in the high-magnification SEM images, appear as flexed platelets of nano-size, and their intersection produces flower-like forms with marked interstitial pores (Figure 2E). This is in agreement with the marked observation according to the HRTEM images, which show the enclaves of the ZA grains randomly inside the blocky matrix of β-CD (Figure 2F). Moreover, the previously reported flower-like forms in the SEM images are detected clearly in the HRTEM images of CD/ZA. 

#### 2.1.3. FT-IR Analysis

**Figure 3:**
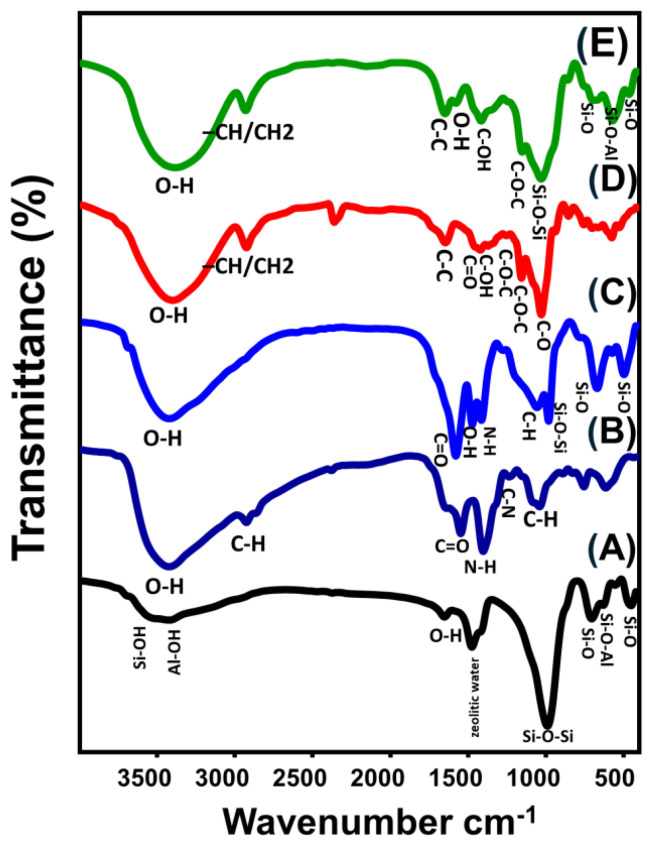
FT–IR spectra of zeolite-A (**A**), chitosan (**B**), CS/ZA composite (**C**), β-CD polymer (**D**), and synthetic CD/ZA composite (**E**).

For the zeolite-A substrate, the main chemical groups of the zeolitic framework were clearly identified from its FT-IR spectrum (Figure 3). These include Si-OH (3612 cm^−1^), Al-OH (3422 cm^−1^), OH stretching (1650 cm^−1^), zeolitic water (1475 cm^−1^), Si-O-Si (990 cm^−1^), Si-O (705 and 452 cm^−1^), and Si-O-Al (630 and 555 cm^−1^) (Figure 3A) [37,38]. N-H (1547 cm^−1^), OH (3423 cm^−1^), C-H (2925 and 1336 cm^−1^), C=O (1637 cm^−1^), C-O (1040 cm^−1^), and C-N (1402 cm^−1^) were distinguished as the essential chemical groups of chitosan (Figure 3B) [26,28]. The spectrum of CS/ZA demonstrates the successful integration and interaction between chitosan and the zeolite chemical structure of ZA (Figure 3C). The identification of chemical groups of both chitosan (C-O (1059 cm^−1^), C-H (1416.7 cm^−1^), and N-H (1582 cm^−1^)) and ZA (zeolitic water (1475 cm^−1^), Si-O-Si (983 cm^−1^), Si-O-Al (574 and 671 cm^−1^), and Si-O (498 cm^−1^)) were detected from its spectrum at remarkable shifted positions (Figure 3C).

Regarding the observed spectrum of β-cyclodextrin demonstrates precisely its basic chemical groups that involve the polysaccharides and glycosidic binding, such as the O-H stretching vibration (3376 cm^−1^), –CH/CH_2_ asymmetrical stretching (2926 cm^−1^), stretching mode of C-C and/or H-O-H deformation within the β-CD cavity (1666.2 cm^−1^), C=O stretching and/or OH bending (1636 cm^−1^), C-OH bending vibration (1482 cm^−1^), symmetrical C-O-C (1200 cm^−1^), asymmetrical C-O-C stretching (1158 cm^−1^), and symmetrical C-O stretching (1000 cm^−1^) (Figure 3D) [1,28]. The obtained spectrum of CD/ZA and the identified bands signify the effective formation of composites from both β-CD and ZA particles (Figure 3E). The main chemical groups of β-CD (asymmetric –CH/CH_2_ (2930 cm^−1^), C-O-C stretching (1152 cm^−1^), and C=O bending (1578 cm^−1^)) were identified in addition to the essential chemical groups of zeolite-A (Al-OH (3388 cm^−1^), zeolitic water (1415 cm^−1^), Si-O-Al (571 and 695 cm^−1^), Si-O-Si (1030 cm^−1^), and Si-O (465 and 858 cm^−1^)) (Figure 3E).

#### 2.1.4. Textural Analysis

The integration influences of the chitosan and β-CD polymers on the textural properties of synthetic zeolite-A were evaluated based on the measured surface area and porosity of the synthetic composites as compared to the individual components. The determined surface area of zeolite-A (423 m^2^/g) was considerably enhanced, to 446.7 m^2^/g and 457.2 m^2^/g, after the hybridization processes with the chitosan (CS/ZA) and β-CD (CD/ZA) polymers, respectively (Table 1). Such an enhancement in the surface area provides a significant high-interaction interface between the surfaces of CS/ZA and CD/ZA as carriers and the dissolved drug molecules during the loading reactions. This was associated with a slight increase in the total pore volumes from 0.382 cm^3^/g to 0.412 cm^3^/g (CS/ZA) and 0.433 cm^3^/g (CD/ZA), in addition to an increase in the average pore diameter (11.6 nm (ZA), 23.6 nm (CS/ZA), and 20.4 nm (CD/ZA)) (Table 1). The previous textural properties were mainly due to the marked changes in the morphological features of ZA particles after the integration of the polymers and the existence of secondary pores related to the matrix of the polymers and the intersections between their grains. 

### 2.2. Encapsulation of 5-Fu

#### 2.2.1. Influence of the Encapsulation Parameters

##### Effect of pH

**Figure 4:**
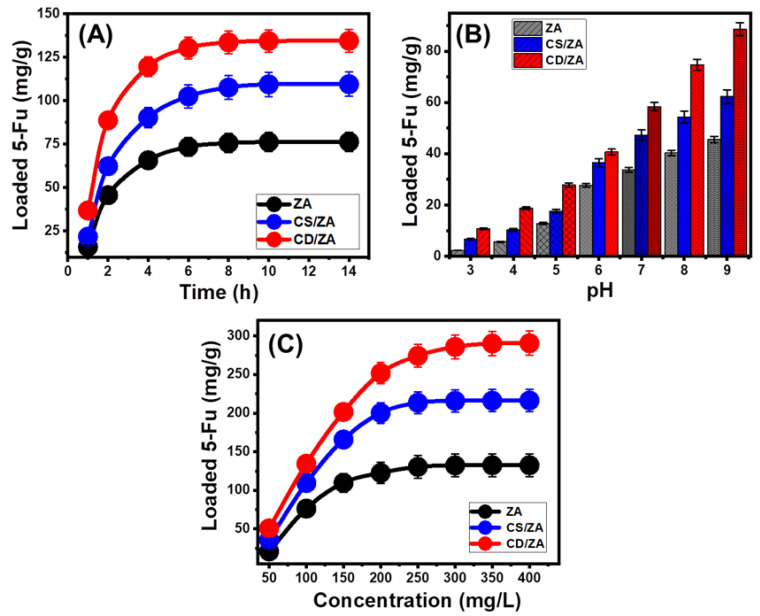
Effect of the main loading variables on the loading capacities of ZA, CS/ZA, and CD/ZA including the effect of pH (**A**), loading duration (**B**), and the tested 5-Fu concentration (**C**).

The effect of the pH of the solutions on the loading capacities of ZA, CS/ZA, and CD/ZA was tested in a range of pH 3 to 9 with fixed levels for the other factors that could affect the results (dose: 20 mg; 5-Fu concentration: 100 mg/L; temperature: 20 °C; volume: 50 mL; time: 2 h). High pH conditions were verified to significantly improve the encapsulation properties of 5-Fu into ZA, CS/ZA, and CD/ZA (Figure 4A). This was detectable from pH 3 (ZA (2.3 mg/g), CS/ZA (6.6 mg/g), and CD/ZA (10.8 mg/g)) to pH 9 (ZA (45.6 mg/g), CS/ZA (62.3 mg/g), and CD/ZA (88.7 mg/g)) (Figure 4A). Therefore, it is advised to load 5-Fu into ZA, CS/ZA, and CD/ZA via encapsulation procedures at basic pH levels. Generally, the adjusted pH of the solutions influences the ionization properties of 5-Fu as well as the dominant surficial charges of ZA, CS/ZA, and CD/ZA. The chemical structure of the 5-Fu medication demonstrates substantial ionization characteristics at higher pH conditions (alkaline) in contrast to its characteristics in acidic to neutral circumstances [11,39]. The 5-Fu ions’ mobility, diffusion, and interactions with the activated and free-loading sites of ZA, CS/ZA, and CD/ZA are strongly influenced by the rise in ionization degrees, which enhance the loading capacities in basic solutions. 

##### Loading Duration 

The effects of the loading period on the capacities of ZA, CS/ZA, and CD/ZA were studied experimentally within a range of 1–14 h, at constant values for all the other influencing variables (pH: 9; 5-Fu concentration: 100 mg/L; temperature: 20 °C; dosage: 20 mg; volume: 50 mL). The 5-Fu loading performances of ZA, CS/ZA, and CD/ZA show considerable improvement in terms of both loading rates and 5-Fu loaded quantities in mg/g with the continuous expansion in the examined time period (Figure 4B). This improvement effect can be recognized from 1 h to 6 h for ZA and CD/ZA and to 8 h for CS/ZA; beyond that, an increase in the test’s duration has a negligible impact either on the detected rate or the loaded amounts of 5-Fu, and the curves show stability states with practically constant values (Figure 4B). These characteristics denote the equilibrium states of ZA, CS/ZA, and CD/ZA as carriers of 5-Fu and attend their equilibration capacities (76.3 mg/g (ZA), 109.5 mg/g (CS/ZA), and 134.5 mg/g (CD/ZA)) (Figure 4B). The observed high loading efficiencies and rapid increase in the 5-Fu loaded quantities may be attributed to the presence of numerous active sites in their free forms on ZA, CS/ZA, and CD/ZA at the beginning of the encapsulating process [38]. As the duration of the tests increases, more and more 5-Fu becomes encapsulated into the preexisting free sites of ZA, CS/ZA, and CD/ZA, leading to occupancy and consumption of these sites, which sharply reduces their availability. As a result, after a particular duration of time, the experimentally measured 5-Fu encapsulating rate obviously decreases, and the 5-Fu loading properties of ZA, CS/ZA, and CD/ZA exhibit little to no enhancement. After all the available sites are occupied by the 5-Fu molecules, the loading equilibrium states of ZA, CS/ZA, and CD/ZA are established [40]. 

##### 5-Fu Concentration

The effects of different concentrations of 5-Fu on the loading capacities of ZA, CS/ZA, and CD/ZA were tested over a range of 50 to 400 mg/L, at fixed values of the other factors (time: 14 h; dose: 20 mg; temperature: 20 °C; pH: 9; volume: 50 mL). The maximal capacities of the ZA, CS/ZA, and CD/ZA carriers, in addition to their equilibrium characteristics, mainly depend on the starting 5-Fu concentrations. In the presence of high 5-Fu starting concentrations, the total quantity of encapsulated 5-Fu in ZA, CS/ZA, and CD/ZA escalated significantly (Figure 4C). As the 5-Fu ions existed in a significantly high concentration at a specific volume, the driving forces and diffusion properties of their ions strongly increased. This increases the opportunity for collisions and promotes chemical interactions between the active sites of ZA, CS/ZA, and CD/ZA and the dissolved ions of the drug [28,40]. This, in turn, increases the 5-Fu loading effectiveness of ZA, CS/ZA, and CD/ZA up to specific concentrations (250 mg/L (ZA and CS/ZA) and 300 mg/L (CD/ZA)) (Figure 4C). Beyond these concentrations, any rise in the assessed 5-Fu concentration has no impact on the measurable loaded quantities of 5-Fu, which usually indicate the equilibrium loading stages of ZA, CS/ZA, and CD/ZA (Figure 4C). As a result, ZA, CS/ZA, and CD/ZA fulfill their maximal 5-Fu loading capacities (132.5 mg/g (ZA), 216.4 mg/g (CS/ZA), and 290.6 mg/g (CD/ZA)). The significantly greater 5-Fu encapsulating capacities of CS/ZA and CD/ZA in comparison with ZA were attributed to a number of factors, including (1) the reported increase in surface area after the modification step, (2) the organophilic properties of CS/ZA and CD/ZA, as compared to the hydrophilic ZA, that promote its affinity to the dissolved organic compounds of 5-Fu, and (3) a substantial rise in the quantities of the existing loading sites after the modification step.

#### 2.2.2. Loading Mechanism 

##### Kinetic Properties

Intra-Particle Diffusion Behavior

**Figure 5:**
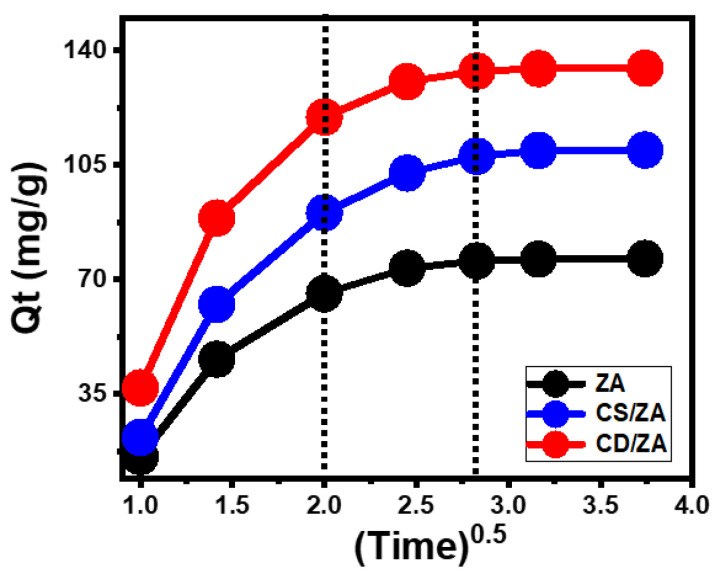
The intra-particle diffusion curves of the 5-Fu loading processes with ZA, CS/ZA, and CD/ZA.

The reactions that encapsulate 5-Fu into ZA, CS/ZA, and CD/ZA display intra-particle diffusion curves with segment-like characteristics, including three distinct phases without crossings with the initial points of the curves (Figure 5). This illustrates 5-Fu’s loading via collaborative processes in addition to the major impact of the drug ions’ diffusion mechanisms towards the active surfaces of ZA, CS/ZA, and CD/ZA [40,41]. This might include (A) loading by the active sites of the exterior surface (border), (B) intra-particle diffusion, and (C) the influence of the saturation or equilibrium stage [42]. The presence of the first stage denotes the activity of the external loading mechanisms at the start of the tests, and the quantity of the surface-active receptors effectively regulates the progress of the encapsulation reactions (Figure 5) [43]. By extending the period, a new stage is identified (Figure 5) that denotes the existence of different controlling mechanisms, including the impact of the layered loading activities as well as the 5-Fu diffusion processes. Finally, the third stage is identified as the dominating phase during the 5-Fu encapsulation equilibrium states of ZA, CS/ZA, and CD/ZA. This verifies the occupancy or consumption of all the active binding sites by the loaded 5-Fu ions (Figure 5) [11,40]. During this step, the loading activities are controlled by a variety of mechanisms that can involve molecular attraction and/or interionic attraction [28].

Kinetic Modeling

**Figure 6:**
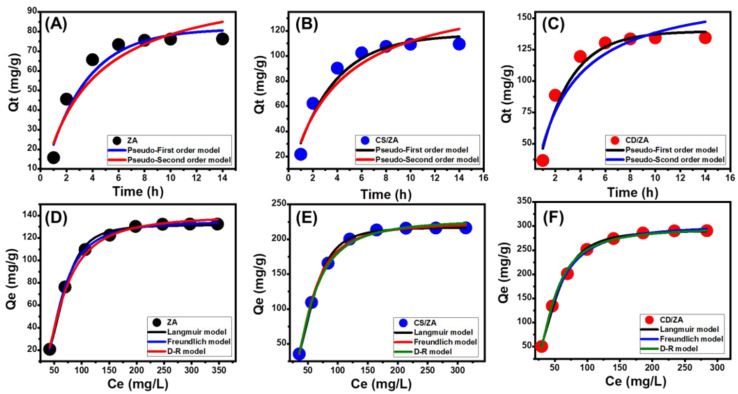
Fitting of the 5-Fu loading processes with the kinetic models (ZA (**A**), CS/ZA (**B**), and CD/ZA (**C**)) and classic isotherm models (ZA (**D**), CS/ZA (**E**), and CD/ZA (**F**)).

Based on the kinetic hypotheses of two different models, a pseudo-first order mode (P.F.) (Equation (1)) and a pseudo-second order (P.S.) (Equation (2)) model, the kinetic characteristics of the process of the encapsulation of 5-Fu into ZA, CS/ZA, and CD/ZA are illustrated. The level of agreement between the loading behaviors and the kinetic assumptions of the two models was evaluated by means of non-linear fitting with their descriptive equations, with the correlation coefficient (R^2^) and chi-squared (χ^2^) as key markers of the fitting degree (Table 2; Figure 6A–C).

(1) $$Q_{t}=Q_{e}\left(1-e^{{-k}_{1}.t}\right)$$

(2) $$Q_{t}=\frac{Q_{e}^{2}k_{2}t}{1+Q_{e}k_{2}t}$$

The determined values of R^2^ and χ^2^ show that the P.F. model’s kinetic characteristics more appropriately represent the 5-Fu loading processes into the ZA, CS/ZA, and CD/ZA than the P.S. model. The striking match between the formerly experimentally identified equilibrium capacities (76.3 mg/g (ZA), 109.5 mg/g (CS/ZA), and 134.5 mg/g (CD/ZA)) and theoretically estimated values from the P.F. model as fitting parameters (81.4 mg/g (ZA), 117.09 mg/g (CS/ZA), and 139.7 mg/g (CD/ZA)) (Table 2) provided further validation for these fitting results. Based on the kinetic characteristics of the representative P.F. model, the loading of 5-Fu into ZA, CS/ZA, and CD/ZA proceeded primarily by physical processes that may have entailed van der Waals forces and/or electrostatic attractions [44,45]. 

However, the loading processes are better described by the P.F. model than by the P.S. model, and the fitting findings are still in substantial agreement with the P.S. model. Consequently, it was anticipated that there would be a minor impact or assisting influence for weak chemical reactions such as hydrogen bonding, electron sharing, hydrophobic interactions, and chemical complexes during the loading of 5-Fu into ZA, CS/ZA, and CD/ZA [40,44]. The collaboration of both physical and chemical processes entailed the production of a chemically loaded layer of the medication, followed by the construction of a physically loaded layer utilizing the first layer as a substrate [46].

##### Isotherm Properties

Classic Isotherm Models

The equilibrium properties of the process loading 5-Fu into ZA, CS/ZA, and CD/ZA as prospective carriers were described utilizing the Langmuir (Equation (3)) and Freundlich (Equation (4)) assumptions, as well as the Dubinin–Radushkevich (D-R) (Equation (5)) hypothesis. The models’ illustrative equations were used to non-linearly fit the findings, and the degree of fit was measured by the value of the correlation coefficient (R^2^) and chi-squared (χ^2^) (Table 2; Figure 6D–F).

(3) $$Q_{e}=\frac{Q_{max}bC_{e}}{\left(1+bC_{e}\right)}$$

(4) $$Q_{e}=K_{f}C_{e}^{1/n}$$

(5) $$Q_{e}=Q_{m}e^{-\beta {ɛ}^{2}}$$

The loading of 5-Fu into ZA, CS/ZA, and CD/ZA exhibits the equilibrium characteristics of the Langmuir isotherm rather than the Freundlich hypothesis, in agreement with the stated values of the model-fitting parameters. As a consequence, it is inferred that the 5-Fu molecules were uniformly trapped on the external surfaces of ZA, CS/ZA, and CD/ZA in monolayer layers via numerous uniformly and homogeneously distributed active sites [11,43]. Furthermore, the preferential encapsulation of 5-Fu ions into ZA, CS/ZA, and CD/ZA carriers was revealed by RL parameter values that were less than one. The theoretical maximum 5-Fu loading capacities of ZA, CS/ZA, and CD/ZA were also calculated as mathematical parameters for the Langmuir isotherm and were determined to be 139.7 mg/g, 220.2 mg/g, and 295.4 mg/g, respectively.

The isothermal properties of the investigated D-R model may shed light on the energy heterogeneity of ZA, CS/ZA, and CD/ZA as carriers of 5-Fu, regardless of the homogeneity or heterogeneity of their respective surface areas [47]. Determining the Gaussian energy (E) as an attainable theoretical parameter of the D-R model emphasizes the nature of the major loading mechanisms, whether chemical or physical in origin. The physical loading process exhibits a Gaussian energy of less than 8 kJ/mol, while the chemical loading process exhibits values > 16 kJ/mol. Gaussian energy values between 8 and 16 kJ/mol are a sign of complex systems or ineffective chemical loading mechanisms [11,47]. The Gaussian energies of the 5-Fu loading processes with ZA, CS/ZA, and CD/ZA are 3.92 kJ/mol, 4.58 kJ/mol, and 5.46 kJ/mol, respectively (Table 2). These values suggest a dominant impact for the physical mechanism during the loading of 5-Fu in addition to the expected effect of zeolitic ion exchange processes (0.6 kJ/mol to 25 kJ/mol).

Advanced Isotherm Models

**Figure 7:**
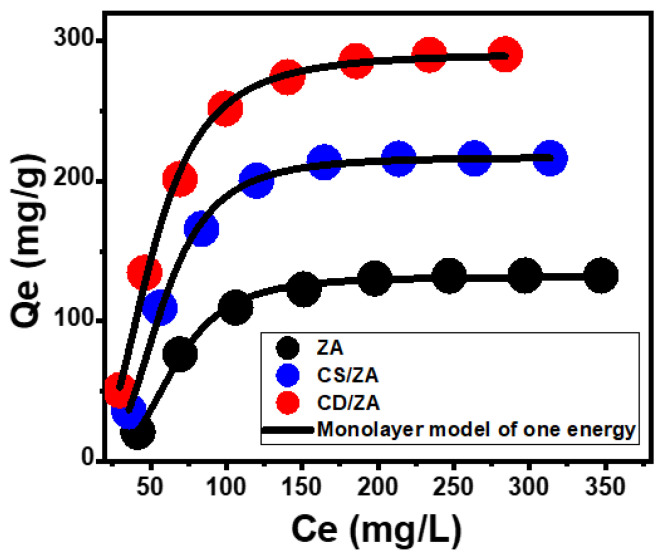
Fitting of the processes of loading 5-Fu into ZA, CS/ZA, and CD/ZA with an advanced monolayer model of one energy site.

The designated advanced isotherm mathematical models, based on the equilibrium fundamentals of statistical physics theory, give further insight into ZA, CS/ZA, and CD/ZA as 5-Fu carriers with regard to the carrier surfaces/drug solution interface. The loading behaviors and their controllable mechanistic activities were studied via an advanced monolayer model with one energy (Equation (6)) and its related theoretical parameters, either steric or energetic (Figure 7; Table 2). The determination coefficients (R^2^) and the root mean square error (RMSE) were determined to be the main factors that determined the fitting degrees.

(6) $$Q=nN_{o}=\frac{nN_{M}}{1+{\left(\frac{C1/2}{Ce}\right)}^{n}}=\frac{Q_{o}}{1+{\left(\frac{C1/2}{Ce}\right)}^{n}}$$

The mathematically investigated steric parameters that were derived from the model comprised the density of the occupied active loading sites (Nm _(5-Fu)_) of ZA, CS/ZA, and CD/ZA, the number of loaded 5-Fu ions per one active site (n _(5-Fu)_), and the 5-Fu loading capacities of ZA, CS/ZA, and CD/ZA at their saturation levels (Qsat _(5-Fu)_). The determined 5-Fu loading energy (E) was the evaluated energetic parameter. The estimated density of the effective loading sites increased significantly as a result of the modifications of the ZA (Nm _(5-Fu)_ = 35.8 mg/g) into CS/ZA (64 mg/g) and CD/ZA (102.7 mg/g). This may have been caused by the incorporation of additional active and free functional groups that are related to hybridized chitosan and β-CD or as a result of the improvement in the interaction interface together with a consequent increase in the surface area. The calculated 5-Fu loading capacities of the ZA, CS/ZA, and CD/ZA at their saturation stages were significantly enhanced after the modification methods, rising from 134.2 mg/g for the ZA to 218.2 mg/g and 291.3 mg/g for the CS/ZA and CD/ZA, respectively. Additionally, the recognized numbers of the loaded 5-Fu ions in each active site of ZA, CS/ZA, and CD/ZA (n _(5-Fu)_) highlight the important influence of the modification processes on the characteristics of the ZA surface as a drug carrier, especially by β-CD. Theoretically, n _(5-Fu)_ is equal to 3.75, 3.4, and 2.8 when 5-Fu is loaded into ZA, CS/ZA, and CD/ZA, respectively. These numbers exceed 1, which suggests the vertical loading of these ions on their exterior surfaces as well as the consequent entrapment of them by multi-molecular mechanisms [48,49]. The loading energies (E) were evaluated using Equation (7) (Table 2) depending on the theoretically derived residual 5-Fu concentrations at the half saturation states (C1/2) and 5-Fu’s solubility in water.

(7) $$∆E=-RTln\left(\frac{S}{C_{1/2}}\right)$$

The determined loading energies of 5-Fu into ZA, CS/ZA, and CD/ZA were −5.43 kJ/mol, −5.84 kJ/mol, and −6.37 kJ/mol, respectively. These values support the previous findings about the physical encapsulation mechanisms (ΔE ≤ 40 kJ/mol) of 5-Fu into ZA, CS/ZA, and CD/ZA [48]. These processes might involve van der Waals forces (ΔE = 4 to 10 kJ/mol), dipole forces (ΔE = 2 to 29 kJ/mol), and hydrogen bonding (ΔE < 30 kJ/mol) [50,51].

### 2.3. In Vitro Release Profiles

**Figure 8:**
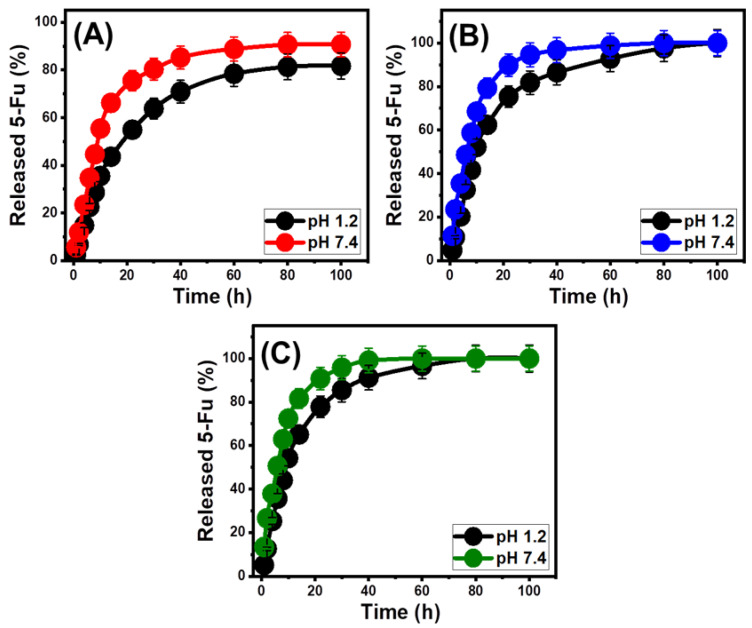
The in vitro release profiles of the 5-Fu drug from ZA (**A**), CS/ZA composite (**B**), and CD/ZA composite (**C**).

The release profiles of ZA, CS/ZA, and CD/ZA were analyzed by measuring the percentages of 5-Fu molecules that moved through gastric fluid (pH 1.2) and intestinal fluid (pH 7.4), which were used to simulate the conditions of cancerous cells (Figure 8). The observed 5-Fu diffusion percentages from ZA, CS/ZA, and CD/ZA with the two investigated buffers show noticeable variations in the recognized rates, with a considerable increase in the estimated release durations (Figure 8A–C). The 5-Fu release rates from ZA, CS/ZA, and CD/ZA display quick characteristics that correlate to significant changes in the measured 5-Fu released quantities. After specific release periods, the detectable 5-Fu diffusion rates significantly decrease, and no appreciable improvement in the released quantities can be observed (Figure 8). By this time, the release reactions of ZA, CS/ZA, and CD/ZA had subsequently stabilized. The quick 5-Fu diffusion properties that were observed during the earliest release periods were attributed to the sudden desorption of the poorly bonded as well as physically loaded 5-Fu ions on the surficial loading sites of ZA, CS/ZA, and CD/ZA [52,53,54]. Following full desorption of such barely bonded and surficially loaded 5-Fu ions, their release characteristics were controlled by the strongly bonded ions or those that formed chemical complexes, as well as the entrapped 5-Fu ions inside the structural pores of ZA, CS/ZA, and CD/ZA, which negatively influenced the observed diffusion rates (Figure 8) [6,20,55]. The strong ionization and solubility properties of 5-Fu in the basic conditions promote the release characteristics of its ions from ZA, CS/ZA, and CD/ZA at pH 7.4 (intestinal fluid) as compared to pH 1.2 (gastric fluid) [56,56]. 

The experimental 5-Fu release patterns of ZA within the gastric as well as intestinal fluids were sustained over 100 h (Figure 8A). After 22 h and 10 h at pH 1.2 and pH 7.4, respectively, approximately 50% of the 5-Fu load had been released from the ZA structure (Figure 8A). Even after 100 h, the entire 5-Fu load had not been released in either the gastric (81.7%) or intestinal fluids (90.6%) (Figure 8A). The predicted strong hydrogen interactions between the loaded 5-Fu ions and the synthetic zeolite’s dominating active hydroxyl-bearing functional groups might explain the low-releasing pattern of ZA [57]. This limits the successful transfer of the medication at the therapeutic level by hindering the liberation of the 5-Fu ions from the zeolite structure. The 5-Fu release profiles of CS/ZA show faster characteristics than the presented profiles of ZA, both at pH 1.2 and pH 7.4 (Figure 8B). After 10 and 8 h at pH 1.2 and pH 7.4, respectively, almost 50% of the loaded amount of 5-Fu seeped from the structure of CS and ZA (Figure 8B). The entire 5-Fu release in both the gastric and intestinal fluids appeared clearly after 100 h and 80 h, respectively (Figure 8B). The hybridization of ZA with β-CD (CD/ZA) also resulted in an increase in the release characteristics of 5-Fu ions (Figure 8C). After 10 h at pH 1.2 and 6 h at pH 7.4, approximately 50% of the 5-Fu had been released from the structure of CD/ZA. After 80 h and 60 h, respectively, the full release of 5-Fu in the gastric and intestinal fluids was detected (Figure 8C).

The observed acceleration in the release speed after the hybridization of ZA with the utilized biopolymers of chitosan and β-cyclodextrin is attributed to their role as coating materials or barriers that exist between the active groups of the zeolite structure and the chemical structure of the 5-Fu drug. This inhibited the predicted hydrogen bonding between the structure of the drug and the hydroxyl-bearing active chemical groups of ZA, causing the 5-Fu molecules to diffuse rapidly. Furthermore, the homogeneous loading of these 5-Fu ions into the matrix of polymer chains has a major impact on the speed of the drug diffusion [58]. Additionally, the incorporation of these polymers introduces extra active sites, increasing the probability of the 5-Fu ions being physically loaded into these surficial sites. In specific situations, during which there is a need for prolonged contact and interaction between the medication ions and the cancerous cells, the gradual and regulated diffusion of 5-Fu as an anticancer medication was advised [5,6]. Additionally, in certain situations requiring specific therapeutic doses to be administered within a short period of time, abrupt and quick delivery methods are recommended. As a result, the synthetic CS/ZA and CD/ZA, as prospective carriers of 5-Fu, may offer a favorable delivery system with regulated encapsulating and release features.

### 2.4. Release Kinetics Studies

Kinetic investigations of the 5-Fu release processes from ZA, CS/ZA, and CD/ZA were conducted as indicators of the properly controlled mechanistic processes. Modeling of the release chemical processes in accordance with zero-order (Z-O) (Equation (10)), first-order (F-O) (Equation (8)), Higuchi (H-G) (Equation (95)), Hixson–Crowell (H-C) (Equation (11)), and Korsmeyer–Peppas (K-P) (Equation (12)) kinetic mathematical models was employed to display the mechanisms on the basis of the linear regression fitting degrees with these models [6].

(8) $$W_{t}-W_{0}=K_{0}.t$$

(9) $$\ln\left(W_{\infty }/W_{t}\right)=K_{1}.t$$

(10) $$W_{t}=K_{h}t^{1/2}$$

(11) $$W_{o}^{1/3}-W_{t}^{1/3}=K_{HC}t$$

(12) $$W_{t}/W_{\infty }=K_{p}t^{n}$$

The zero-order kinetic characteristics indicate the occurrence of the release processes at constant rates and without a significant effect of the loaded doses on the releasing effectiveness of 5-Fu from ZA, CS/ZA, and CD/ZA [4]. In terms of F-O release kinetics, the doses of 5-Fu loaded into ZA, CS/ZA, and CD/ZA have a significant impact on the release efficiency [1]. The kinetic hypothesis of Higuchi kinetics (H-G) implies that the diffusion mechanisms have a predominant effect on the release systems [1,59]. The diffusion processes based on Higuchi kinetics were performed at a constant rate that was less than the loaded quantities of 5-Fu. Furthermore, the employed carriers must have sink characteristics, and the influence of their swelling and solubility on their release behaviors is ignored [4]. The Hixson–Crowell model’s (H-C) kinetic assumption depends on erosion mechanisms rather than diffusion, and the reactions that occur according to its kinetic hypothesis display a significant impact of the surface area and grain diameter of the used carriers on the release processes [4,27]. According to the mechanistic assumption of Korsmeyer–Peppas kinetics, the release mechanisms entail the cooperation of diffusion and erosion mechanisms [1,60].

**Figure 9:**
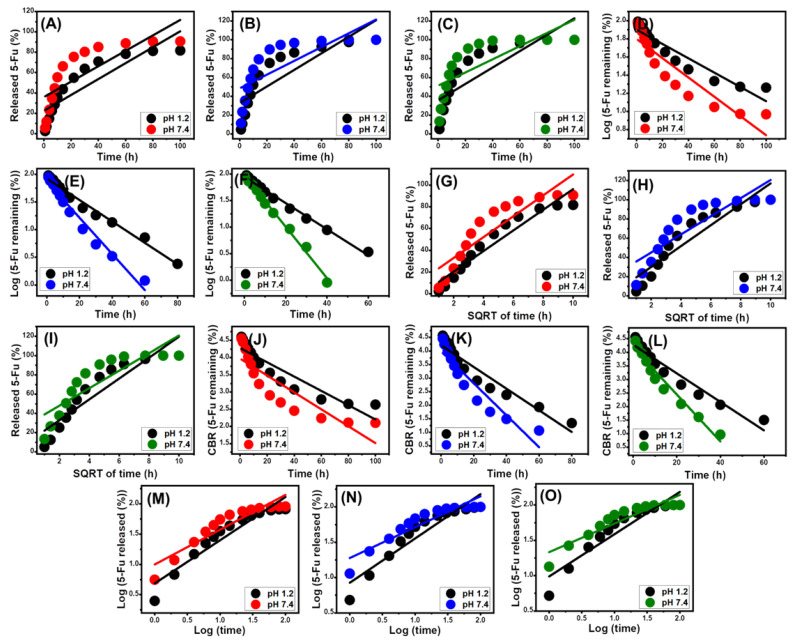
Fitting of the 5-Fu release results with a zero-order model (ZA (**A**), CS/ZA (**B**), and CD/ZA (**C**)), first-order model (ZA (**D**), CS/ZA (**E**), and CD/ZA (**F**)), Higuchi model ((ZA (**G**), CS/ZA (**H**), and CD/ZA (**I**)), Hixson–Crowell model (ZA (**J**), CS/ZA (**K**), and CD/ZA (**L**)), and Korsmeyer–Peppas model (ZA (**M**), CS/ZA (**N**), and CD/ZA (**O**)).

Based on the determination coefficients (R^2^), the reported 5-Fu release processes of ZA, CS/ZA, and CD/ZA mimic the characteristics of the F-O (Figure 9D–F; Table 2) kinetics rather than the Z-O kinetics (Figure 9A–C; Table 2), indicating the substantial impact of the loaded 5-Fu quantities on the release efficiency. The release reactions demonstrate excellent consistency with both the Higuchi (H-G) (Figure 9G–I; Table 2) and Hixson–Crowell (H-C) (Figure 9J–L; Table 2) models. These kinetic evaluation data revealed that diffusion and erosion mechanisms collaborated during the 5-Fu release events. However, the Hixson–Crowell kinetics are more closely followed by the release patterns of CS/ZA and CD/ZA, and these characteristics are primarily influenced by erosion processes. The reported substantial fitting degrees of the release behaviors with the Korsmeyer–Peppas kinetics and the calculated values of the diffusion exponent (n) as a fitting parameter (Figure 9M–O; Table 2) confirmed the complex mechanistic hypothesis. The diffusion exponent (n) values are greater than 0.45, indicating that the release reactions of the ZA, CS/ZA, and CD/ZA delivery systems have non-Fickian transport features [13].

### 2.5. Cytotoxicity Properties

**Figure 10:**
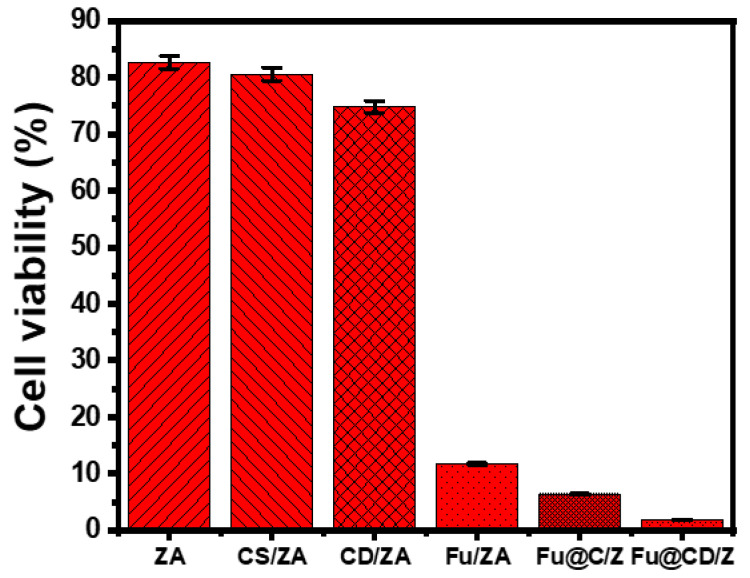
The cytotoxicity effect of free ZA, CS/ZA, and CD/ZA particles and their 5-Fu-loaded products on colorectal cancer cells (HCT-116).

The cytotoxicity of free ZA, CS/ZA, and CD/ZA, as well as their 5-Fu-loaded derivatives, was tested on both fresh colorectal fibroblast cells (CCD-18Co) and colorectal cancer cells (HCT-116). The established cytotoxic effects of ZA, CS/ZA, and CD/ZA as free particles on normal CCD-18Co cells show that they are safe and compatible enough to be used as recommended drug carriers, taking into account the range of doses tested (20–120 g/L). The determined cell viability of fresh cells in the presence of ZA, CS/ZA, and CD/ZA at their maximum doses (120 µg/L) is 90.6%, 91.8%, and 94.3%, respectively. Regarding the cytotoxicity of ZA, CS/ZA, and CD/ZA as free particles against the HCT-116 cancer cells tested, the products have strong cytotoxicity against tumor cells, especially at the highest doses (500 µg/L), with cell viability values of 82.7%, 80.6%, and 76.8%, respectively. This means that the investigated carriers inhibit cancerous cells by 17.3% (ZA), 19.4% (CS/ZA), and 23.2% (CD/ZA). In terms of the cytotoxicity and anticancer potential of 5-Fu-loaded ZA, CS/ZA, and CD/ZA against the evaluated HCT-116 tumor cell lines, the 5-Fu-loaded products were better than the free 5-Fu medication as a positive control. The cell viability, IC-50, and inhibiting percentage of ZA (500 g/mL) were determined to be 11.72%, 127.3 g/mL, and 88.28%, respectively (Figure 10). According to the observed findings for the produced CS/ZA, its anticancer effects have been significantly improved (5.43% (cell viability), 9.56 g/mL (IC-50), and 94.57% (inhibitory percentage)) (Figure 10). The measured values for the 5-Fu-loaded CD/ZD composite also notably improved to 1.83% (cell viability), 98.17% (inhibitory %), and 4.16 g/mL (IC-50) (Figure 10). These results demonstrate a significant improvement in the cellular cytotoxicity and anticancer properties following the loading of the 5-Fu drug into the investigated carriers, particularly the hybridized products of zeolite-A (CS/ZA and CD/ZA), in addition to the previously established regulating effects on the loading and release behaviors.

### 2.6. Comparison Study

The 5-FU loading properties of ZA as well as its modified forms (CS/ZA and CD/ZA) as inorganic and hybrid delivery systems were compared with other investigated structures in the literature (Table 3). As can be observed, the polymeric functionalized products display higher loading capacities than natural (clinoptilolite) and synthetic zeolites (zeolite-A, HY zeolite), clay-based carriers (Montmorillonite/magnetite composite, Magadiite-CTAB-chitosan), synthetic nanoporous silicate, and its composite with chitosan. Such findings validate the significant effect of the polymeric modification processes on enhancing the loading properties of zeolite-A, either by inducing surface reactivity or increasing the active loading sites. Moreover, this signifies the promising qualifications of zeolite-A to be applied as a delivery system of enhanced capacity after facile modification processes as compared to natural clay-based structures and several synthetic materials. 

## 3. Experimental Work

### 3.1. Materials

The kaolinite powder that was used during the production of the zeolite was obtained directly from the Central Metallurgical Research & Development Institute, Egypt. β-cyclodextrin polymer (>85%), ethanol (95%), acetic acid (99.8%), and chitosan powder (MW 120,000; 85%) were all obtained from Sigma-Aldrich, Egypt, as analytical-grade products and applied during the hybridization processes. 5-fluorouracil (analytical grade >99%) was obtained from Sigma-Aldrich, Egypt, to be used during the investigated loading, release, and cytotoxic experiments.

### 3.2. Synthesis of Chitosan/Zeolite-A (CS/ZA) and β-cyclodextrin/Zeolite-A (CD/ZA)

The zeolite-A synthesis operations were carried out using the approach described by Shaban et al. [35]. The kaolinite powder was activated thermally to obtain metakaolinite by a heating process for 4 h at 750 °C. Following that, the metakaolinite product underwent homogenization in a NaOH solution for 12 h while stirring at a metakaolinite/NaOH weight ratio of 1:2. The resulting aluminosilicate gel was subsequently placed in a Teflon-lined stainless steel autoclave and underwent hydrothermal treatment for 4 h at 150 °C. The synthetic products were filtered, washed to neutralize the particles, and then left to dry at 70 °C overnight.

The fabrication of the chitosan/zeolite-A composite (CS/ZA) was synthesized according to the reported procedures of Jiang et al. [28]. Approximately 4 g of zeolite particles was dispersed within 100 mL of distilled water and subjected to 180 min of sonication treatment. This was subsequently mixed with the previously prepared solution of chitosan (4 g of chitosan dissolved in 100 mL of acetic acid (0.1 M)). The slurry was then efficiently homogenized using a sophisticated mixing method that included 12 h of sonication and 800 rpm magnetic stirring. Following that, the product was filtered, carefully rinsed to prevent the negative effects of the remaining acetic acid, and dried slowly at 60 °C for 12 h. The prepared composite was identified as CS/ZA and was kept for further experimental procedures.

For the production of the β-cyclodextrin/zeolite-A hybrid, 4 g of synthesized zeolite was homogenized thoroughly with 100 mL of distilled water utilizing a magnetic stirring device (500 rpm) and an ultrasound source (240 W) over 60 min. A comparable experiment included dissolving 4 g of the β-CD with 100 mL of ethanol and then homogenizing this mixture for 60 min. Following that, an ultrasound source (240 W) was used to mix the β-CD solution together with the obtained zeolite-A suspension, and the resultant mixture was agitated for 24 h. Following the mixing period, the β-CD/ZA particulates were separated from the sample by centrifuging it for 15 min at 3000 rpm. After thorough washing with distilled water, the obtained composite was slowly dried at 60 °C for 12 h. Finally, the product was labeled as CD/ZA and used in the other experimental steps.

### 3.3. Analytical Techniques

The degree of crystallinity and the present crystal phases were measured employing a PANalytical-Empyrean X-ray diffractometer over a detection range of 0 to 70° based on the resulting XRD patterns. The chemical structures of ZA, CS/ZA, and CD/ZA were distinguished using a Fourier transform infrared spectrometer (FTIR8400S; Shimadzu) within the detection frequency spectrum from 400 cm^−1^ to 4000 cm^−1^. SEM photos were obtained using a scanning electron microscope (Gemini, Zeiss Ultra 55) immediately after coating ZA, CS/ZA, and CD/ZA with thin gold layers. The predicted changes in the morphological properties of zeolite after the two different modification steps were confirmed using the obtained SEM images. Additionally, the inner characteristics of the ZA, CS/ZA, and CD/ZA were studied utilizing HRTEM images, which were obtained by a transmission electron microscope (JEOL-JEM2100) at an accelerating voltage of 200 kV. The surface area and porosity of the ZA, CS/ZA, and CD/ZA were determined using a surface area analyzer (Beckman Coulter SA3100) and the related N_2_ adsorption/desorption isotherms.

### 3.4. 5-Fu Loading Studies

The investigations encapsulating 5-Fu into ZA, CS/ZA, and CD/ZA were assessed based on the essentially addressed aspects to regulate the 5-Fu encapsulated dose as well as its greatest encapsulation capacities. The pH (3–9), encapsulation time (1–14 h), 5-Fu concentration (50–400 mg/L), and temperature (20–60 °C) were the main parameters that were evaluated during the study. The ZA, CS/ZA, and CD/ZA particles were efficiently homogenized inside the tested 5-Fu aqueous solutions (50 mL) using a vortex rotator. Following each test’s equilibration duration, the ZA, CS/ZA, and CD/ZA particles were extracted from the 5-Fu solutions by filtering them via Whatman paper, and the remaining 5-Fu concentrations were then determined using a UV–vis spectrophotometer at an adjusted wavelength (λ _(max)_ = 266 nm). The remaining 5-Fu concentrations were employed to compute the loading capacities of ZA, CS/ZA, and CD/ZA in mg/g based on Equation (13). The experiments loading 5-Fu into ZA, CS/ZA, and CD/ZA were performed in triplicate, and the calculated average values were provided in the research data with standard deviations of 3.8%.

(13) $$\mathrm{Loaded}\mathrm{drug}\left(mg/g\right)=\frac{\left(\mathrm{Initial}\mathrm{concentration}-\mathrm{Residual}\mathrm{concentration}\right)\times \mathrm{solvent}\mathrm{volume}}{\mathrm{Carrier}\mathrm{weight}}$$

### 3.5. The Release Studies

The 5-Fu release patterns of the ZA, CS/ZA, and CD/ZA materials were assessed in two different chemical buffers (gastric fluid, pH 1.2, and intestinal fluid, pH 7.4) at 37.5 °C. The 5-Fu-loaded ZA, CS/ZA, and CD/ZA particles (100 mg/g) were extensively dispersed individually throughout 500 mL of the evaluated release buffers. The loaded samples were prepared according to the estimated best loading conditions (time: 14 h; dose: 20 mg; temperature: 20 °C; pH: 9; volume: 50 mL; concentration: 100 mg/L). The DISTEK dissolving apparatus homogenized the 5-Fu-loaded ZA, CS/ZA, and CD/ZA particles and the two distinct buffers for 120 h at 200 rpm as the adjusted vessel’s rotational speed. To monitor the 5-Fu diffusion percentages from ZA, CS/ZA, and CD/ZA, a UV–vis spectrophotometer was employed to analyze samples of the two different buffered solutions (5 mL), which were taken at periodic intervals from the bulk volumes of the release solutions. The bulk release buffers were immediately replenished with the regularly collected samples to keep the volumes at exactly the same levels during the entire release time. The 5-Fu release experiments were completed in triplicate, and the calculated average results were given in the studies using Equation (14) with a standard deviation of less than 4.21%.

(14) $$\mathrm{Drug}\mathrm{release}\left(\%\right)=\frac{\mathrm{The}\mathrm{amount}\mathrm{of}\mathrm{Released}5-\mathrm{Fu}}{\mathrm{Amount}\mathrm{of}\mathrm{loaded}5-\mathrm{Fu}}\times 100$$

### 3.6. In Vitro Cytotoxicity

#### 3.6.1. Cell Lines

Colorectal cancer cell lines (HCT-116) were delivered from the American Type Culture Collection (ATCC, Rockville, MD) and assessed as the target cancer cells during the conducted cytotoxic assays. Gentamycin, 0.25% trypsin-EDTA, fetal bovine serum, HEPES buffer, dimethyl sulfoxide (DMSO), RPMI-1640, 3(4, 5-dimethylthiazol-2-yl)-2.5 diphenyltetrazolium bromide (MTT 99%), and DMEM are the essential chemical reagents that were employed during the performed incubation process and cytotoxic assays. All the incubation processes and the cytotoxicity assays were accomplished at the Regional Center for Mycology and Biotechnology, Al-Azhar University, Egypt.

#### 3.6.2. In Vitro Cytotoxicity

RPMI-1640 medium combined with 50 g/mL gentamycin and 10% fetal calf serum was first used to cultivate the malignant HCT-116 cell lines at 37 °C and 5% CO_2_. The malignant cell lines (5 × 10^4^ cells/well) had been cultured for three weeks before being immersed in Corning^®^ 96-well plates for 24 h. Then, particular dosages of the 5-Fu-loaded CS/ZA and CD/ZA were administered to the cell strains which were then cultured for a further 24 h. The loaded samples were prepared according to the estimated best loading conditions (time: 14 h; dose: 20 mg; temperature: 20 °C; pH: 9; volume: 50 mL; concentration: 100 mg/L). The number of viable cells generated throughout the duration of incubation was determined using the widely employed MTT cell proliferation assessment. The incorporated culture medium was efficiently removed by finishing the incubation cycle and replaced with newly generated media (100 µL of RPMI). The freshly added media was mixed thoroughly with the MTT (10 µL; 12 mM), and the combination was then cultured once more for 5 h to see whether formazan, with a distinguishable purple hue, had grown. The generated formazan was then successfully dissolved using 50 µL of DMSO solution. In the last stage, a microplate set to a particular wavelength of 590 nm was used to measure the optical densities (ODs) of the cell lines that were cultivated during the investigations. According to Equation (15), the computed values were utilized to determine the cell viability percentage [11].

(15) $$\mathrm{Cell}\mathrm{viability}\left(\%\right)=\frac{\mathrm{Mean}\mathrm{OD}}{\mathrm{Control}\mathrm{OD}}\times 100$$

## 4. Conclusions

Synthetic zeolite-A was functionalized effectively with chitosan and β-cyclodextrin producing two forms of biocomposites (CS/ZA and CD/ZA). The two products were applied as enhanced carriers of the 5-Fu drug with enhanced loading properties (218.2 mg/g (CS/ZA) and 291.3 mg/g (CD/ZA)) compared to ZA (134.2 mg/g). Classical and advanced equilibrium modeling revealed that CD/ZA has a higher density of 5-Fu active loading sites (102.7 mg/g) than both CS/ZA (64 mg/g) and ZA (35.8 mg/g). By predominantly physical multi-molecular mechanisms, each active loading site of these carriers may hold three to four vertically oriented 5-Fu ions. The 5-Fu release profiles of CS/ZA and CD/ZA may last as long as 80 h and are characterized by non-Fickian transport characteristics with complex diffusion and erosion mechanisms. Concerning their cytotoxic impacts on HCT-116 cancer cell lines, the 5-Fu-loaded samples had cell viability % of 11.72% (ZA), 5.43% (CS/ZA), and 1.83% (CD/ZA).

## Figures and Tables

**Table 1:** Textural properties of zeolite-A, CS/ZA, and CD/ZA based on the determined results by a surface area analyzer (Beckman Coulter SA3100) and the obtained related N_2_ adsorption/desorption isotherms.

Sample	Specific Surface Area	Total Pore Volume	Average Pore Size
Zeolite-A	423 m^2^/g	0.382 cm^3^/g	11.6 nm
CS/ZA composite	446.7 m^2^/g	0.412 cm^3^/g	23.6 nm
β-CD/ZA composite	457.2 m^2^/g	0.433 cm^3^/g	20.4 nm

**Table 2:** The obtained mathematical parameters of the studied kinetic, classic isotherm, advanced isotherm, thermodynamic, and release kinetics models.

Model	Parameters			ZA	CS/ZA	CD/ZA
Kinetic Models						
Pseudo-first order	K_1_ (min^−1^) *Qe*_(*Cal*)_ (mg/g) R^2^ X^2^			0.32 81.46 0.95 0.99	0.301 117.09 0.95 1.25	0.402 139.7 0.96 0.94
Pseudo-second order	k_2_ (g mg^−1^ min^−1^) *Qe*_(*Cal*)_ (mg/g) R^2^ X^2^			0.0025	0.0016	0.0021
				107.12	155.8	174.7
				0.91 1.55	0.92 1.94	0.92 1.8
Isotherm models						
Langmuir	*Q*_*max* (mg/g)_*b* (L/mg)R^2^X^2^RL			139.7 2.18 × 10^−7^ 0.99 0.07 0.99	220.2 1.09 × 10^−6^ 0.99 0.065 0.99	295.4 1.46 × 10^−5^ 0.99 0.147 0.99
Freundlich	1/n k_F_ (mg/g) R^2^ X^2^			0.65 3.58 0.99 0.11	0.61 7.47 0.99 0.13	0.59 12.1 0.99 0.214
D-R model	β (mol^2^/kJ^2^) *Q*_*m*_ (mg/g) R^2^ X^2^ E (kJ/mol)			0.03246 140.3 0.99 0.12 3.92	0.0238 228.4 0.99 0.11 4.58	0.0167 296.3 0.99 0.12 5.46
Monolayer model of one energy	n Nm (mg/g) *Q*_(*sat*)_ (mg/g) ∆E (kJ/mol)			3.75 35.8 134.2 −5.43	3.41 64 218.2 −5.84	2.83 102.7 291.3 −6.37
**Release kinetics**						
Models				Determination coefficient		
	ZA		CS/ZA		CD/ZA	
	Acetate buffer (pH 1.2)	Phosphate buffer (pH 7.4)	Acetate buffer (pH 1.2)	Phosphate buffer (pH 7.4)	Acetate buffer (pH 1.2)	Phosphate buffer (pH 7.4)
Zero order	0.74	0.59	0.67	0.52	0.65	0.49
First order	0.89	0.81	0.97	0.96	0.98	0.99
Higuchi	0.91	0.83	0.86	0.80	0.85	0.80
Hixson–Crowell	0.86	0.76	0.91	0.86	0.93	0.95
Korsmeyer–Peppas	0.90	0.85	0.88	0.85	0.87	0.85
n	0.71	0.57	0.62	0.47	0.59	0.48

**Table 3:** Comparison between the loading capacities of the studied carriers and other carriers in literature.

Carrier	Loading Capacity (mg/g)	Reference
Calcium silicate biocomposite	12.3	[61]
Magadiite-CTAB-chitosan	162.29	[62]
Montmorillonite/magnetite	59.44	[14]
Magadiite	98.18	[62]
Magadiite-CTAB	130.59	[62]
Chitosan/MCM-48	191	[20]
Montmorillonite	90	[63]
Ca-montmorillonite	23.3	[64]
Clinoptilolite	138.9	[11]
HY zeolite	110	[64]
ZA	134.2	This study
CS/ZA	218.2	This study
CD/ZA	291.3	This study

## Data Availability

Data are available upon reasonable, by the Corresponding Authors.
